# Assessment of carotid artery invasion by lymph node metastasis from squamous cell carcinoma of aero-digestive tract

**DOI:** 10.1016/S1808-8694(15)30755-2

**Published:** 2015-10-19

**Authors:** Abrão Rapoport, Olger de Souza Tornin, Ivo Marques Beserra Júnior, Paulo Bentes de Carvalho Neto, Ricardo Pires de Souza

**Affiliations:** 1 Livre docente habilitation, Sao Paulo University (USP) Medical School. Technical director of the health department, Heliopolis Hospital; 2 Master in health science, Heliopolis Hospital graduate program, HOSPHEL/SP, Radiologist; 3 Graduate student, health science course, Heliopolis Hospital, HOSPHEL/SP; medical resident, Heliopolis Hospital, HOSPHEL/SP; 4 Graduate student, health science course, Heliopolis Hospital, HOSPHEL/SP; medical resident, Heliopolis Hospital, HOSPHEL/SP; 5 Doctor, Sao Paulo University (USP) Medical School. Deputy coordinator of the graduate program on health science, Heliopolis Hospital, HOSPHEL/SP. Radiologist. Otorhinolaryngology and Head & Neck Surgery, and Radiology Departments, Heliopolis Hospital, HOSPHEL, Sao Paulo, Brazil

**Keywords:** carotid artery, squamous cell carcinoma, lymph node, metastasis, computed tomography

## Abstract

Carotid involvement by metastatic lymph nodes changes the approach in head and neck tumors.

**Aim:**

To evaluate interobserver agreement by CT scan analysis regarding the involvement of the carotid artery by metastatic lymph nodes from squamous cell carcinoma of the upper aerodigestive tract and its relation to resection criteria. Materials and

**Methods:**

retrospective study of 99 CT images of patients with SCC were evaluated. Eighty-six were males and 13 females, with ages ranging from 32 to 76 years. Four radiologists, without any previous knowledge of the clinical stage, analyzed imaging results. No patients had received previous treatment and histological diagnoses were obtained through biopsy. The carotid artery invasion was classified as simple (0 to 50% and from to 100%), and complex (0 to 25%; 26 to 50%; 51 to 75% and 76 to 100%). The level of interobserver agreement was obtained through Kappa Index (p&8804; 0, 05) and the concordance power varied from despicable to excellent.

**Results:**

The Kappa Index were moderate (0, 53%) for simple classification and minimum (0, 36%) for complex classification.

**Conclusions:**

The computed tomography showed low effectiveness in the evaluation of lymph node metastasis resection concerning carotid artery invasion.

## INTRODUCTION

Lymph node involvement affects the choice of therapy, given that metastatic carotid artery invasion usually implies in non-surgical treatment. In this situation, surgical morbidity and mortality rates are high,^1^ reducing 5-year survival by up to 50%.^2^^,^^3^

Before computed tomography (CT), assessing cervical lymph node and carotid artery involvement was restricted to the physical examination of the neck and to intraoperative findings.^4^ The diagnosis of upper aerodigestive tract carcinoma metastases (with or with no rupture of the capsule) became more accurate with the advent of sectional image methods such as CT and magnetic resonance imaging (MRI),^5^, ^6^, ^7^ improving survival rates by up to 50%.^8^, ^9^, ^10^

Consequently, it is important to learn beforehand whether the carotid arteries are surrounded or not by lymph node metastases; this finding might redefine a lesion as being non-resectable, or involve prior planning of complex carotid artery grafting techniques.

The aim of this paper was to assess the interobserver reproducibility in evaluating the extent of carotid artery involvement by metastatic lymphadenomegaly.

## METHOD

A retrospective study (1990 to 2004), approved by the Research Ethics Committee (number 394), was done to assess CT exams of the neck of 99 patients diagnosed with upper aerodigestive tract carcinoma. Primary lesions were in the mouth (12), nasopharynx (2), oropharynx (30), hypopharynx (22), larynx (23) and hidden primaries (10). All of the patients presented epidermoid carcinoma metastatic lymphadenomegaly, demonstrated by histopathology.

There were 13 female patients (13.1%) and 86 male patients (86.9%) aged between 32 and 76 years (mean – 55.6 years; median – 57 years). Smoking was reported in 71 patients (71.7%) and alcohol drinking was reported in 64 patients (64.6%); all of the patients that consumed alcohol were also smokers. Patients were placed in dorsal decubitus, and axial CT images (5.0/5.0 mm slice thickness/increment) were acquired from the suprasellar area to the superior portion of the sternoclavicular joint, angulating the gantry perpendicular to the aerodigestive tract. Iodinated contrast (1.0 to 2.0 ml/kg at 60% and 76%) was injected endovenously in all patients.

Four radiologists trained in head and neck image diagnosis (five years' experience each) evaluated the exam results. They were named observer 1 (observ 1), observer 2 (observ 2), observer 3 (observ 3), and observer 4 (observ 4). The four examiners assessed the exams individually, having no prior information of the clinical staging, and applied criteria for evaluating the extension of lesions. The radiologists filled in their reports according to two classification systems, based on their judgment of the degree of circumferential carotid artery involvement: a simplified two-item classification (0–50%, and 51–100% involvement) and a complex four-item classification (0–25%, 26–50%, 51–75%, and 76–100% involvement). These had been extrapolated initially from CT assessments of aortic involvement in esophageal carcinoma cases,^11^ and used later in the MRI assessment of carotid involvement.^12^

**Figure 1:**
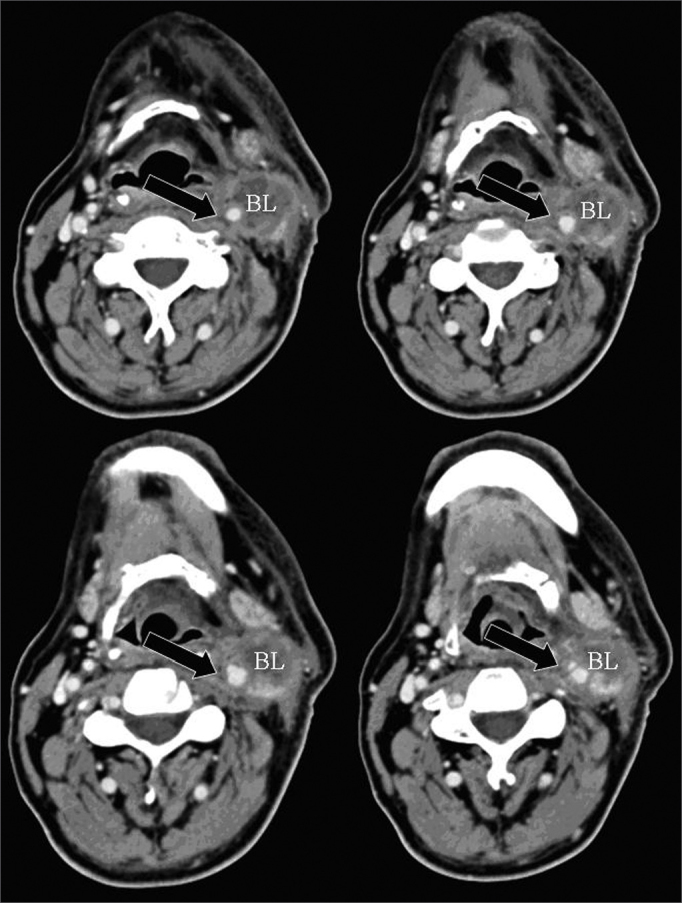
axial CT with endovenous contrast. Lymphadenomegaly / high jugular-carotid (level II) lymph node block (BL) to the right with a contact interface below 50% of the internal carotid artery circumference. CI: internal carotid artery; CE: external carotid artery.

**Figure 2:**
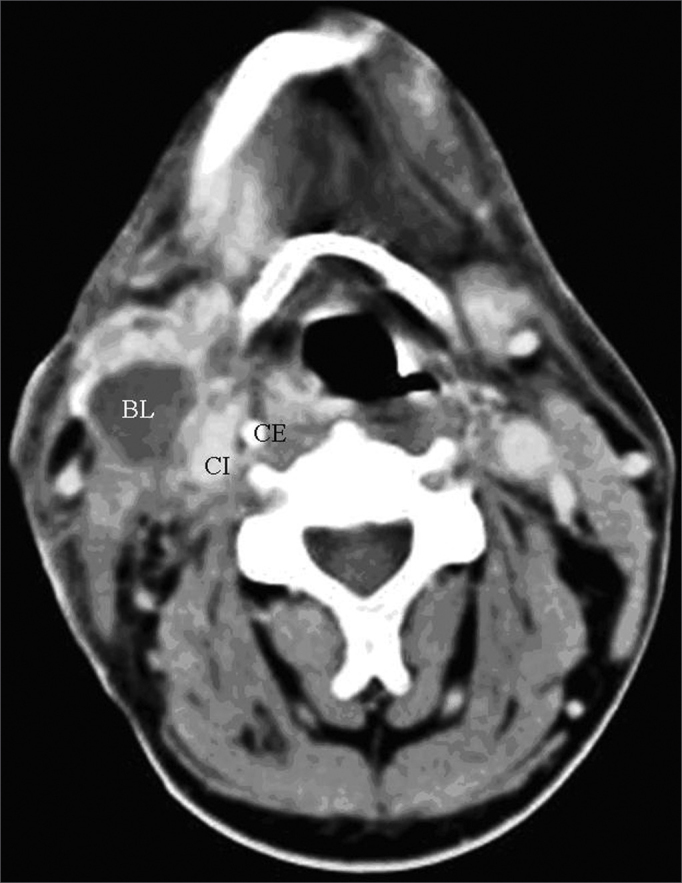
axial CT with endovenous contrast. – high jugular-carotid (level II) lymph node block (BL) to the left with a contact interface below 50% of the internal carotid artery circumference. CI: internal carotid artery; CE: external carotid artery.

**Figure 3:**
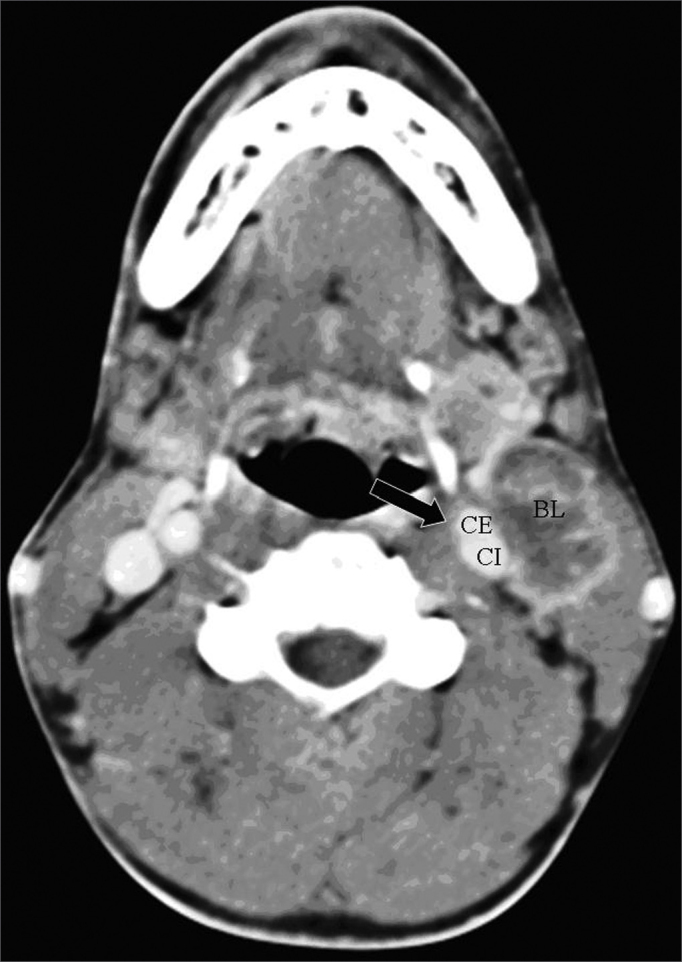
axial CT (4 sequential sections) with endovenous contrast. High jugular-carotid (level II) lymph node block (BL) to the left with signs of rupture of the capsule and a contact interface over 50% of the internal carotid artery circumference (arrow).

One of the CT signs of lymph node involvement was enlargement of lymph nodes (diameter over 1.5 cm) in the axial plane only (Figure 1). A rounded morphology was also suspect, as the usual lymph node shape is oval. Two or more adjacent lymph nodes grouped within the same lymph node plane (Figure 2) and involving 75% of the internal carotid artery circumference were named lymph node clusters. Another important criterion was central necrosis, which frequently presented as contrast rim enhancement of lymph nodes. An irregular interface between lymph node margins and its surrounding fat or muscle was also an important criterion for suspecting rupture of the capsule (Figure 3).

The degree of carotid artery involvement was the contact interface between the enlarged lymph node considered as metastatic and the circumference of the vessel (0–25%, 26–50%, 51–75%, and 76–100%).

Radiological findings were confirmed by surgical and pathological findings in 27 of 99 patients.

### Statistical method

After CT exam interpretation, the Kappa (k) index was used for assessing interobserver agreement; Frame 1 shows the agreement criteria at a p=0.05 significance level and a 95% confidence interval.

**Frame 1:** Assessment of interobserver agreement according to the calculated Kappa index

KAPPA INDEX VALUE	AGREEMENT
< 0,20	NEGLIGIBLE
0,21 – 0,40	MINIMAL
0,41 – 0,60	MODERATE
0,61 – 0,80	GOOD
0,81 – 1,0	EXCELLENT

In those cases that presented bilateral lymph node involvement, “n” was doubled, considering both sides (right and left) as separate samples. The statistical software was the EPIDAT, version 1.0, 1994, Junta de Galicia, PAHO.

## RESULTS

TNM 2002 clinical staging was as follows: T1 (4), T2 (19), T3 (32), and T4 (32); there were also 10 cases (10.1%) diagnosed as hidden primaries (Tx), and 2 cases (2.0%) whose stage was not defined in their files. Regarding lymph node involvement, 69 cases were clinically staged as N2 (a, b, or c) and N3 (69.8% of patients). Another 28 cases were staged N1 and N0 (28.2%). Staging was not reported in the files of 2 cases (2.0%).

There was no vascular invasion in any of the patients that were operated and whose surgical specimens were studied by the pathology lab (27 patients). In our sample, this means a negative predictive value of 100% for carotid artery wall invasion by metastatic cervical lymph nodes.

Table 1, Table 2, Table 3, Table 4 show the interobserver agreement values using both classifications (simplified and complex) in assessing carotid artery involvement by metastatic cervical lymph nodes from upper aerodigestive tract epidermoid carcinomas.

**Table 1:** Result of observer assessment in absolute numbers (simplified classification)

	Observer 1	Observer 2	Observer 3	Observer 4
0–50%	96	102	105	102
51–100%	30	24	21	24
Total	126	126	126	126

**Table 2:** Interobserver agreement – simplified classification

	Kappa*	Confidence interval**	P***	Agreement
0 – 50%	0,535	0,46 – 0,60	< 0,0001	Moderate
51 –100%	0,535	0,46 – 0,60	< 0,0001	Moderate

* general Kappa = 0.535

** CI = Confidence interval – 95%; sup. 0.606; inf. 0.464

*** p < 0.001

**Table 3:** Result of observer assessment in absolute numbers (complex classification)

	Observer 1	Observer 2	Observer 3	Observer 4
0–25%	61	47	37	51
26–50%	35	55	68	51
51–75%	8	19	13	13
76–100%	22	5	8	11
Total	126	126	126	126

**Table 4:** Interobserver agreement – complex classification

	Kappa*	Confidence interval**	P***	Agreement
0–25%	0,499	0,42–0,57	<0,0001	Moderate
26–50%	0,294	0,22–0,36	<0,0001	Minimal
51–75%	0,206	0,13–0,27	<0,0001	Negligible
76–100%	0,362	0,29–0,43	<0,0001	Minimal

* general Kappa = 0.364

** CI = Confidence interval – 95%; sup. 0.412; inf. 0.316

*** p < 0.001

## DISCUSSION

This study aimed to evaluate interobserver agreement in analyzing CT images of carotid artery involvement by metastatic cervical lymph nodes from upper aerodigestive tract epidermoid carcinomas; two classifications are used (simplified and complex).^11^, ^12^, ^13^

A possible bias in the agreement evaluation method may occur if the radiologists have different experience levels, as has been observed in previous studies.^14^^,^^15^ In this study, therefore, radiologists with similar experience in head and neck radiology – trained at the same image diagnosis unit – were sought.

Many papers^4^, ^5^, ^6^^,^^12^^,^^16^, ^17^, ^18^ have described CT as the best method for image-based staging of cervical lymph nodes. These studies compared image findings with pathology lab findings, obtaining good sensitivity and specificity values in staging cervical lymph nodes, and consequently, their relation with the carotid artery.

Various statistical methods may be used to measure and explain the total variation; part of this variation is random, part is due to method deficiencies, and part results from observer failure.^19^^,^^20^ Reproducibility and the negative predictive value may be evaluated, the latter based on cases that progressed to surgical resection. There was no vascular invasion in such cases among our sample, yielding a 100% negative predictive value. Reproducibility is the ability of a measurement or observation to yield the same or a similar result to those measurements or observations of the same fact obtained by other methods or by medical interpretation.^19^^,^^20^

Statistically significant values were obtained in two classification systems from the analysis of interobserver variation in CT image analysis of carotid artery involvement by metastatic cervical lymph nodes from upper aerodigestive tract epidermoid carcinomas.

Few papers^14^^,^^15^^,^^21^ have been published on interobserver agreement in staging upper aerodigestive tract carcinomas; most of these papers did not refer to the status of lymph nodes involved; this is described below.

Good agreement was achieved for the jugular-carotid chains in all exams. In those cases where lymph nodes are very close to the internal jugular vein doubts may arise about whether they belong to the jugular-carotid chain. This becomes evident in jugular-carotid chains associated with a more medial sternocleidomastoid muscle. This, however, does not affect significantly the treatment strategy; if lymph node dissection is chosen, the surgical approach will be lateral.^22^ Our results for this series revealed that, when using CT, there is moderate interobserver agreement in the simplified classification and negligible interobserver agreement in the complex classification.

These results are very useful for clinical practice, as the general kappa index for the simplified classification (0.53) is close to the critical acceptance level, which is 0.6. As for agreement in the complex classification, the general kappa was 0.36, which is minimal agreement. Low agreement in the complex classification may be due to the increased number of intervals, which may have jeopardized the analysis when close to the borderline between intervals; a further factor is the diameter of the carotid artery, which is lower than that of the descending aorta.^11^ At intervals below 50% contact with the carotid artery, for instance, the prognosis is reasonable; this was the case of most of the patients that were operated in our series. Carotid artery involvement over 180 degrees has a worse surgical prognosis.^23^ Involvement over 270 degrees does not by itself mean carotid wall invasion – as we observed in our series – since inflammation may coexist with lymphadenomegaly. On the other hand, a published paper on carotid artery involvement over 270 degrees assessed by MRI indicated that wall invasion was present in 83% of cases.^12^

Based on patient files, only the negative predictive value could be calculated, as only 27 patients were operated. In these resected cases, the negative predictive value was 100%; the pathology reports were present in all of their files. There were no descriptions of carotid artery invasion.

Surgeons decide whether to operate or not based on clinical and image data. Placing prostheses in place of the invaded carotid artery is becoming less common, as morbidity and mortality rates are significantly high with this procedure. Even peeling procedures have been attempted as therapeutic alternatives when involvement is not more than 180 degrees.^23^ This does not mean, however, that surgeons – when analyzing CT images and finding involvement over 270 degrees – do not assess the possibility of intervening with curative aims in young patients with mobile tumors and good clinical status. Frequently, in such cases, the final decision may be taken based on intraoperatory findings or even during salvage surgery.

The CT report should be used as a further criterion for understanding the difficulties that surgeons may face, but never for predicting potentially curative procedures, given that this method tends to overestimate arterial involvement.^24^ This was the case in the analyses made by observer 1 compared to the other observers. Even when there are huge lesions, involvement is not considered if periarterial fat is not altered; this may have been the cause of error made by observer 1, given that most of the lesions in the sample were classified as being in advanced stages.

In lymphadenomegalies that displaced the carotid artery, observer 1 tended to consider this situation as being of high involvement degree; by itself, this is incorrect, as the other three observers reported.

Based on this study with four observers, we suggest using the simplified classification; not only does it increase the agreement between radiologists, but it also avoids overestimating the degree of involvement, which tends to occur when using the complex classification. This would avoid dissuading those professionals that might have indicated or implemented curative treatment and that would end up choosing palliative measures.

## CONCLUSION

The general kappa was 0.53 in the simplified classification (moderate agreement) and 0.36 in the complex classification (minimum agreement) for interobserver agreement on CT image analysis of carotid artery involvement by metastatic cervical lymph nodes from upper aerodigestive tract epidermoid carcinomas.
